# γδTFH cells promote B cell maturation and antibody production in neuroblastoma

**DOI:** 10.1186/s12865-017-0216-x

**Published:** 2017-07-07

**Authors:** Wenjun Mou, Wei Han, Xiaoli Ma, Xiaolin Wang, Hong Qin, Wen Zhao, Xiaoya Ren, Xi Chen, Wei Yang, Haiyan Cheng, Xisi Wang, Hui Zhang, Xin Ni, Huanmin Wang, Jingang Gui

**Affiliations:** 1 Key Laboratory of Major Diseases in Children, Ministry of Education, Beijing Children’s Hospital, Capital Medical University, National Center for Children’s Health, Beijing, 100045 China; 2 Laboratory of Immunology, Beijing Pediatric Research Institute, Beijing Children’s Hospital, Capital Medical University, National Center for Children’s Health, Beijing, 100045 China; 3 Department of Surgical Oncology, Beijing Children’s Hospital, Capital Medical University, National Center for Children’s Health, Beijing, 100045 China; 4 Hematology Oncology Center, Beijing Children’s Hospital, Capital Medical University, National Center for Children’s Health, Beijing, 100045 China; 5 Department of Otolaryngology, Head and Neck Surgery, Beijing Children’s Hospital, Capital Medical University, National Center for Children’s Health, Beijing, 100045 China

**Keywords:** Neuroblastoma, γδT cells, CXCR5, Interleukin 4, Interleukin 10, B cells

## Abstract

**Background:**

Previous studies have shown that γδ TFH cells are capable of modulating antibody production in immunized and infected mouse model. In recent studies, human γδ TFH cells are shown to contribute to the activation of humoral immunity and promote the maturation of B cells. However, little information is available on their involvement in neuroblastoma (NB) pathogenesis.

**Results:**

In the present study, the frequency of γδ TFH cells in 74 NB patients was significantly higher compared with that in 60 healthy controls. Moreover, most γδ TFH cells in NB patients had a naive phenotype with up-regulation of CD25, CD69, HLA-DR and CD40L and down-regulation of ICOS. Importantly, γδ TFH cells in NB patients produced more IL-4 and IL-10 than those in healthy controls. Furthermore, serum total IgG level was significantly increased in NB patients compared with healthy controls. The expression of CD23 on B cells was up-regulated while CD80 expression was significantly down-regulated in NB patients. Further analysis of B cell compartment showed that the frequency of CD19^+^CD27^hi^ plasma cells was enhanced in NB patients. Spearman’s correlation analysis revealed that the frequency of γδ TFH cells was positively correlated to serum total IgG level and CD19^+^CD27^hi^ plasma cells in NB patients, but negatively correlated to CD19^+^ B cells.

**Conclusions:**

We concluded that γδ TFH cells might promote B cell maturation and antibody production in NB patients.

## Background

The T follicular helper cells (TFH) play a central role in humoral immunity [1]. Besides CD4 TFH cells, natural killer T (NKT) cells, CD8 T cells and γδT cells also involve in humoral immune responses and provide B cell help [2].

The majority of γδT cells in human peripheral blood could recognize non-peptide tumor-associated phospho-antigens which can elicit humoral immune response [3, 4]. Previous studies have shown that γδ TFH cells are capable of modulating antibody production in immunized and infected mouse model [5]. In recent studies, human γδ TFH cells are shown to contribute to the activation of humoral immunity and promote the maturation of B cells [6, 7]. However, little information is available on their involvement in neuroblastoma (NB) pathogenesis.

In the present study, patients diagnosed of NB were analyzed for the percentage and phenotype of γδ TFH cells and their contribution to B cell functions in peripheral blood. We showed here that γδ TFH cells secreted higher level of IL-4 and IL-10 in NB patients than those in healthy controls. Moreover, γδ TFH cells resulted in a substantial increase in the production of serum total IgG antibodies, strongly suggesting that these cells are highly efficient in providing B-cell help for antibody production.

## Methods

### Subjects

A total of seventy-four patients (36 boys, 38 girls; mean age 3.2 ± 0.3 years) with NB were enrolled between January 2014 and July 2016 from Beijing Children’s Hospital. Nineteen individuals with other blastoma (9 boys, 10 girls; mean age 2.8 ± 0.3 years) and sixty age- and sex-matched healthy children (36 boys, 24 girls; mean age 3.1 ± 0.5 years) were recruited as control groups. The study has been approved by ethnics committee of Beijing Children’s Hospital in accordance with principles of the Declaration of Helsinki. Written consent of research purpose was signed by parents or legal guardians of all participants.

### Sample collection

Peripheral blood samples were collected in BD Vacutainer™ plastic blood collection tubes containing EDTA K2 as anticoagulant. Serum was obtained by centrifugation at 3500 rpm for 7 min. PBMCs were separated by standard Ficoll-Hypaque density centrifugation at 1000 RCF for 20 min.

### Flow cytometry

Phenotypic analysis was performed using 100 μl peripheral blood samples. Cells were stained with fluorochrome-conjugated anti-human CD3 (UCHT1), CD19 (HIB19), CD25 (BC96), CD45RA (HI100), CD45RO (UCHL1), CD62L (DREG-56), CD23 (EBVCS-5), CD154 (24-31), CCR7 (G043H7), ICOS (C398.4A), IgD (IA6-2), TCRγδ (B1) (all from Biolegend, San Diego, CA, USA) and anti-human CD27 (M-T271), CD40 (5C3), CD69 (FN50), CD80 (L307.4), CD86 (FUN-1), CXCR5 (RF8B2), HLA-DR (G46-6) (all from BD Biosciences, San Diego, CA, USA). Data were collected by flow cytometry on a FACScalibur and were analyzed with FlowJo software (TreeStar).

### Intracellular staining

PBMCs were stimulated with 5 ng/ml IL-2 (Cell Signaling), 50 ng/ml PMA (Merck), 1 μg/ml ionomycin (Sigma Aldrich), and GolgiStop (BD Biosciences) was added for the final 5 hours. PBMCs were stained with anti-human TCRγδ and CXCR5. PBMCs were then fixed using a BD Perm/Fix intracellular staining kit. PBMCs were then stained with IL-4 (MP4-25D2), IL-10 (JES3-9D7), IFNγ (4S.B3) (all from Biolegend, San Diego, CA, USA) and IL-2 (MQ1-17H12, BD Biosciences, San Diego, CA, USA) at room temperature for 30 min at dark. Data were collected by flow cytometry on a FACScalibur and were analyzed with FlowJo software (TreeStar).

### Measurement of IL-4 and IL-10

Serum IL-4 and IL-10 were measured by Luminex Multiplex assay (Merck) on manufacturer’s instructions.

### Measurement of serum total IgG, IgA and IgM

Serum immunoglobulin (IgG, IgA, IgM) were determined by automated Beckman Immage 800 Immunochemistry System (Beckman Coulter) on manufacturer’s instructions.

### Statistical analysis

All statistical analyses were performed using SPSS 17.0 software and Prism 5.01 software. Two-tailed student t test was used for comparisons between two groups, and One-way ANOVA was used for analysis of differences in three groups. Correlations between variables were determined using Spearman’s correlation coefficient. Statistically significant levels are indicated as follows: *P < 0.05, **P < 0.01, and ***P < 0.001.

## Results

### γδ TFH cells were significantly increased in the peripheral blood of patients with NB

**Fig. 1:**
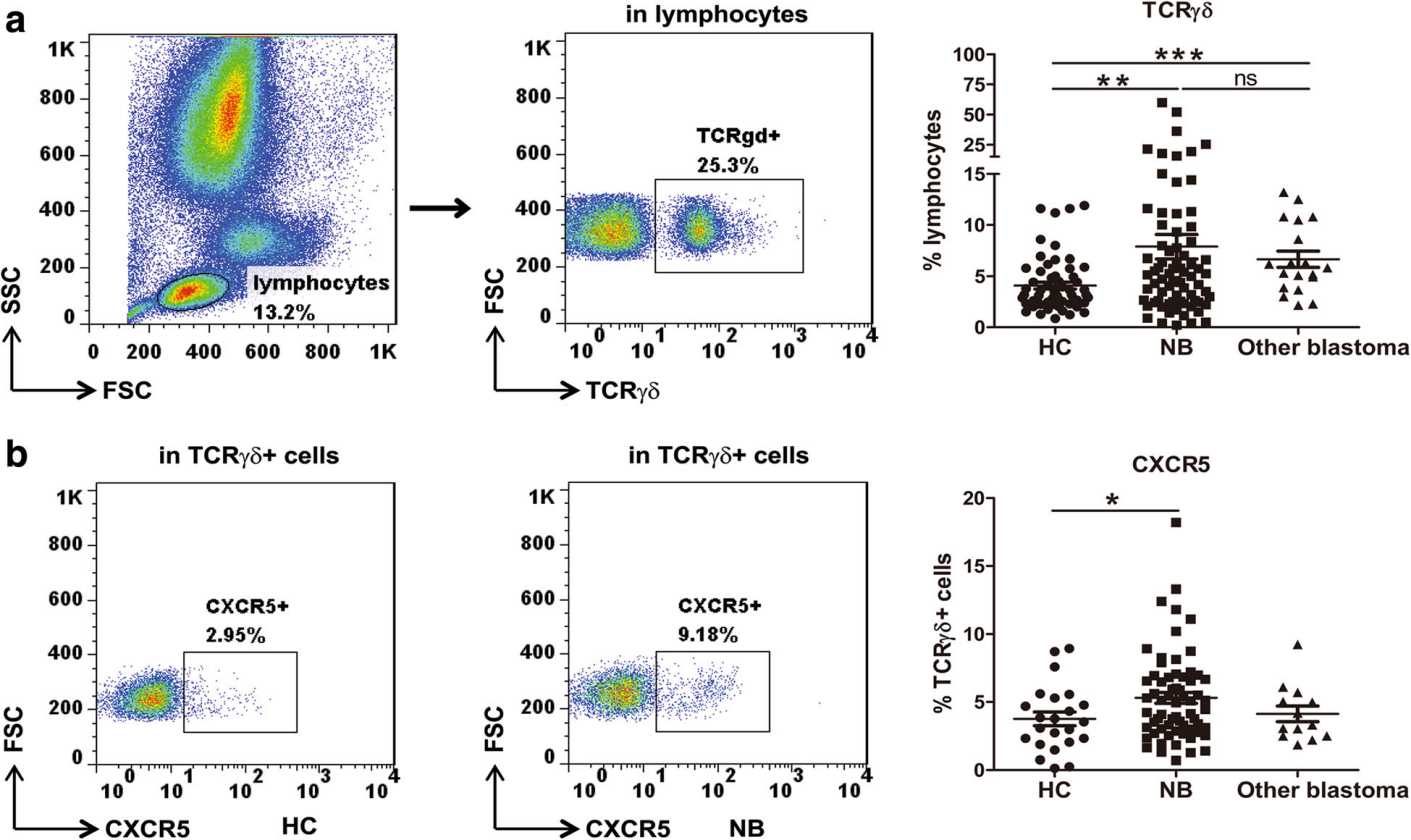
Circulating CXCR5^+^ γδT cells were significantly increased in NB patients. **a** The percentage of γδT cells in peripheral blood from NB patients (*n* = 74), other blastoma patients (*n* = 19) and healthy controls (*n* = 60) were analyzed by flow cytometry. **b** The percentage of CXCR5^+^ cells in γδT cells from NB patients (*n* = 65), other blastoma patients (*n* = 13) and healthy controls (*n* = 19) were analyzed by flow cytometry. Each dot represents one individual. *P < 0.05. **P < 0.01, ***P < 0.001, ns = not significantly different

Using flow cytometry, we detected the frequency of circulating γδT cells in the peripheral blood from 74 NB patients, 19 other blastoma patients and 60 healthy controls. As shown in Fig. 1a, a significantly increased frequency of circulating γδT cells was found in NB patients compared with healthy controls (4.1% ± 0.3 vs 7.9% ± 1.2, *p* = 0.006). There was also a significantly increased frequency of circulating γδT cells in patients with other blastoma compared with healthy controls (4.1% ± 0.3 vs 6.7% ± 0.8, *p* < 0.001, Fig. 1a).

γδ TFH cells can be specified by unique expression of CXCR5 on γδT cells [1]. We then analyzed the expression of CXCR5 on peripheral blood γδT cells from NB patients, other blastoma patients and healthy controls. We found that the frequency of CXCR5^+^ T cells in peripheral blood γδT cells was significantly higher in NB patients compared with healthy controls (3.8% ± 0.5 vs 5.3% ± 0.4, *p* = 0.04, Fig. 1b).

### Phenotypic and functional analysis of γδ TFH cells in NB patients

**Fig. 2:**
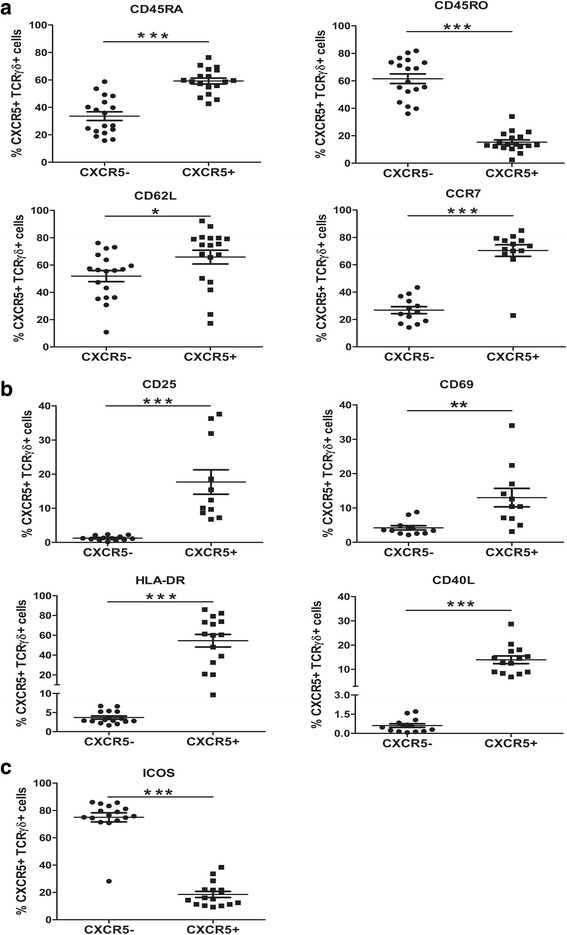
Surface phenotype of γδ TFH cells in NB patients. **a** The percentage of CD45RA, CD45RO, CD62L and CCR7 in CXCR5^+^ γδT cells and CXCR5^−^ γδT cells from NB patients were shown. **b** The percentage of CD25, CD69, HLA-DR and CD40L in CXCR5^+^ γδT cells and CXCR5^−^ γδT cells from NB patients were shown. **c** The percentage of ICOS in CXCR5^+^ γδT cells and CXCR5^−^ γδT cells from NB patients were shown. Each dot represents one individual. *P < 0.05. **P < 0.01, ***P < 0.001

We tested the relative subgroups (naive and memory) in CXCR5^+^ γδT cells and CXCR5^−^ γδT cells in NB patients. FACS analysis demonstrated that the vast majority of peripheral blood CXCR5^+^ γδT cells were CD45RA^+^ but not CD45RO^+^, and expressed higher CD45RA, CCR7 and CD62L (CD45RA, 33.6% ± 3.2 vs 58.7% ± 2.2, *p* < 0.0001; CD45RO, 61.5% ± 3.5 vs 15.3% ± 1.7, *p* < 0.0001; CCR7, 26.8% ± 2.6 vs 70.4% ± 4.3, *p* < 0.0001; CD62L, 51.8% ± 4.1 vs 65.8% ± 5.0, *p* = 0.04, Fig. 2a) compared with CXCR5^−^ γδT cells, suggesting that most of them have a naive phenotype in NB patients.

We then assessed the expression of activation markers and costimulatory molecules on CXCR5^+^ γδT cells. Peripheral blood CXCR5^+^ γδT cells expressed higher activation markers (CD25, 1.3% ± 0.2 vs 17.7% ± 3.6, *p* < 0.0001; CD69, 4.2% ± 0.6 vs 13.0% ± 2.7, *p* = 0.003; HLA-DR, 3.7% ± 0.4 vs 54.5% ± 6.4, *p* < 0.0001, Fig. 2b) and costimulatory molecules (CD40L, 0.6% ± 0.1 vs 14.0% ± 1.6, *p* < 0.0001, Fig. 2b) compared with CXCR5^−^ γδT cells. The expression of ICOS was strongly concentrated to CXCR5^−^ γδT cells with minimal expression in CXCR5^+^ γδT cells (ICOS, 75.0% ± 3.4 vs 18.5% ± 2.2, *p* < 0.0001, Fig. 2c).

**Fig. 3:**
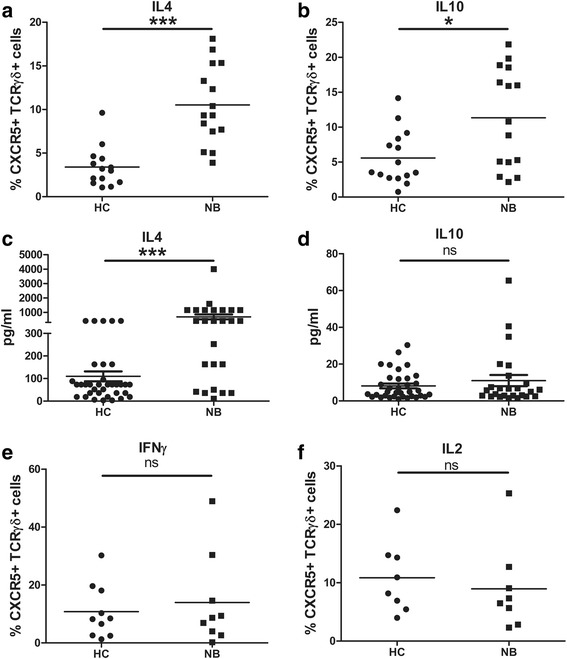
γδ TFH cells secreted IL-4 and IL10 was increased in NB patients. **a** Intracellular staining of IL-4 in CXCR5^+^ γδT cells in NB patients (*n* = 15) and health controls (*n* = 14). **b** Intracellular staining of IL-10 in CXCR5^+^ γδT cells in NB patients (*n* = 15) and health controls (*n* = 15). **c** Serum level of IL-4 were measured by Luminex Multiplex assay in NB patients (*n* = 35) and health controls (*n* = 25). **d** Serum level of IL-10 were measured by Luminex Multiplex assay in NB patients (*n* = 35) and health controls (*n* = 25). **e** Intracellular staining of IFNγ in CXCR5^+^ γδT cells in NB patients (*n* = 10) and health controls (*n* = 9). **f** Intracellular staining of IL-2 in CXCR5^+^ γδT cells in NB patients (*n* = 8) and health controls (*n* = 8). Each dot represents one individual. *P < 0.05. ***P < 0.001, ns = not significantly different

We then studied the pattern of cytokine production in γδ TFH cells. As shown in Fig. 3a, b, CXCR5^+^ γδT cells in NB patients produced more IL-4 and IL-10 than those in healthy controls (IL-4, 3.4% ± 0.6 vs 10.5% ± 1.2, *p* < 0.001, Fig. 3a; IL-10, 5.6% ± 1 vs 11.3% ± 1.8, *p* < 0.05, Fig. 3b). The serum level of IL-4 and IL-10 were also assessed by Luminex Multiplex assay. Serum level of IL-4 was significantly increased in NB patients compared with healthy controls (109.9 ± 21.8 vs 682.6 ± 170.2, *p* < 0.001, Fig. 3c) while there was no significant difference in serum IL-10 between patients with NB and healthy controls (8.1 ± 1.3 vs 11.1 ± 3.0, *p* = 0.3299, Fig. 3d). We then checked IFNγ and IL-2 production by γδ TFH cells. As shown in Fig. 3e, f, there was no significant difference in IFNγ and IL-2 production by γδ TFH cells between patients with NB and healthy controls (IFNγ, 10.8% ± 2.9 vs 14.0% ± 5.3, *p* = 0.594, Fig. 3e; IL-2, 10.9% ± 2.2 vs 9.0% ± 2.6, *p* = 0.584, Fig. 3f).

### Serum total IgG level was increased in NB patients

**Fig. 4:**
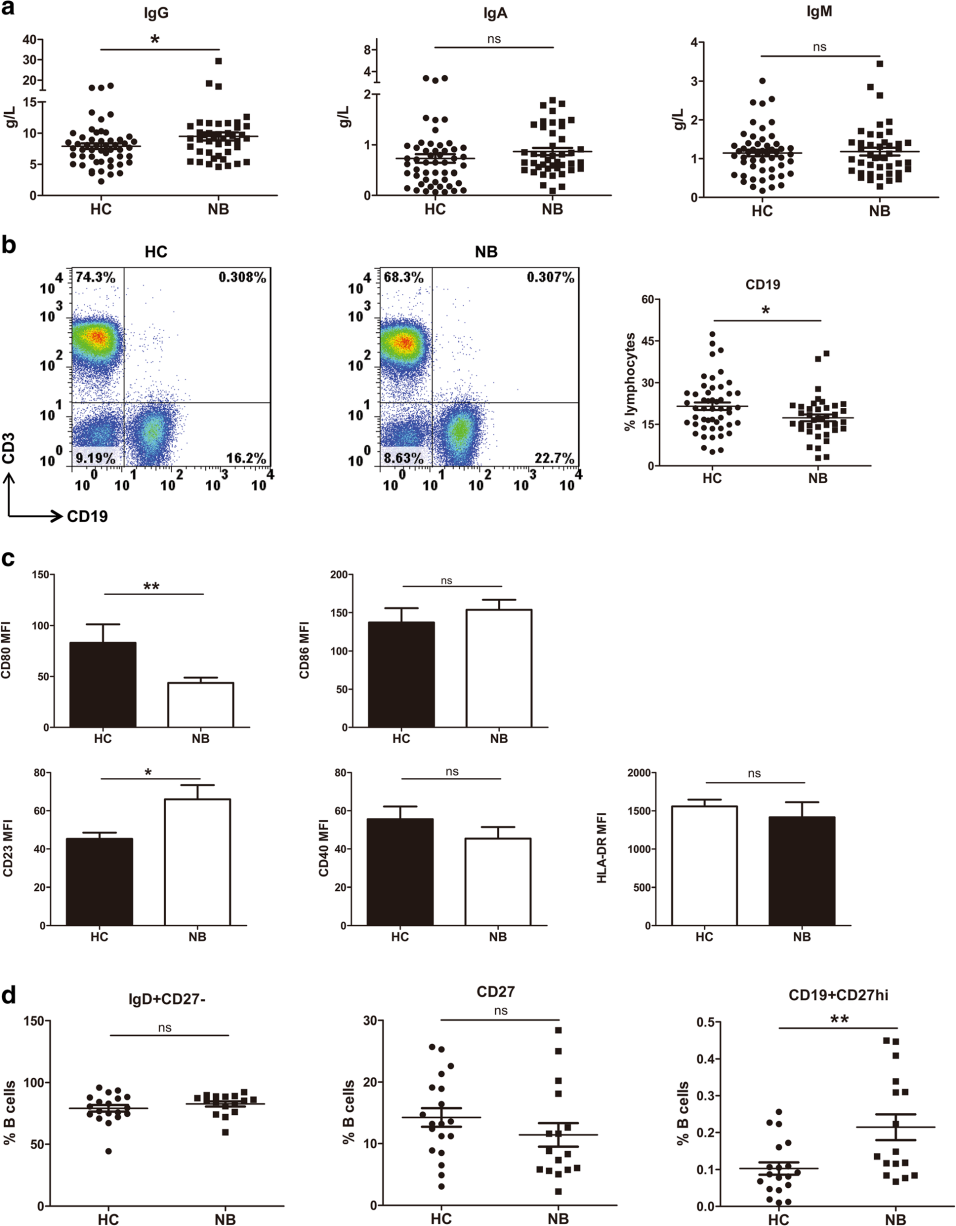
Antibody production and B-cell phenotype in NB patients. **a** Total serum level of IgG, IgA and IgM were measured by ELISA in NB patients (*n* = 43) and health controls (*n* = 52). Each dot represents one individual. *P < 0.05, ns = not significantly different. **b** The percentage of CD3^−^CD19^+^ B cells in peripheral blood from NB patients (*n* = 39) and health control (*n* = 50) were analyzed by flow cytometry. Each dot represents one individual. *P < 0.05. **c** Phenotype analysis of CD3−CD19+ B cells. Data were expressed as the mean + SEM. *P < 0.5, **P < 0.01, ns = not significantly different **d** The percentage of IgD^+^CD27^−^, CD27^+^ and CD19^+^CD27^hi^ B cells in peripheral blood from NB patients and health control were shown. Each dot represents one individual. **P < 0.01, ns = not significantly different. **d** Phenotype analysis of CD3^−^CD19^+^ B cells. Data were expressed as the mean + SEM. **P < 0.01, ns = not significantly different

γδT cells may have a modulatory effect in the control of humoral immune response [7]. Antibodies are major components of humoral immunity, we then assess serum total IgA, IgG and IgM levels in NB patients. Serum total IgG level was significantly increased in NB patients compared with health controls (mean 9.5 ± 0.7 vs 7.9 ± 0.4 g/L, *p* < 0.05, Fig. 4a) while there was no significant difference in serum IgA and IgM between patients with NB and healthy controls (IgA, mean 0.9 ± 0.1 vs 0.7 ± 0.1 g/L, *p* = 0.23; IgM, mean 1.2 ± 0.1 vs 1.1 ± 0.1 g/L, *p* = 0.79, Fig. 4a).

To examine whether increased total serum IgG level in NB patients was a result from an elevation in B cells, we then detected the frequency of circulating CD3^−^CD19^+^ B cells in NB patients. However, the frequency of CD3^−^CD19^+^ B cells decreased in NB patients compared with healthy controls (17.8% ± 1.6 vs 21.6% ± 0.8, *p* = 0.03, Fig. 4b).

### B cells presented with mature phenotype in NB patients

IgG antibodies are generated following activation and maturation of B cells [8]. We then assessed the expression of the activation and maturation markers on B cells in NB patients. As shown in Fig. 4c, the expression of CD23 on B cells were up-regulated in NB patients (MFI: 45.3 ± 3.2 vs 66.0 ± 7.5, *p* = 0.03) while CD40 and HLA-DR expression was not changed in NB patients (MFI: HLA-DR, 1557.0 ± 89.5 vs 1415.0 ± 197.1, *p* = 0.48; CD40, 55.6 ± 6.6 vs 45.5 ± 6.0, *p* = 0.32). In contrast, CD80 expression was significantly down-regulated (MFI: 83.0 ± 18.2 vs 43.7 ± 5.1, *p* < 0.01) while CD86 expression was not changed in NB patients (MFI: 137.3 ± 18.4 vs 153.6 ± 13.1, *p* = 0.51). These data suggest that B cells in NB patients exhibited mature phenotypes with up-regulated CD23 and down-regulated CD80.

### Enhanced plasma cells in NB patients

IgG molecules are created and released by plasma cells [9, 10]. Upon antigen encounter in the periphery, some mature B cells may differentiate to antibody-secreting plasma cells [11, 12]. As we observed increased serum total IgG level and a mature phenotype in NB patients, we then assess the frequency of CD19^+^CD27^hi^ plasma cells in NB patients. As shown in Fig. 4d, the frequency of CD19^+^CD27^hi^ plasma cells were increased in NB individuals compared with health controls (0.1% ± 0.02 vs 0.2% ± 0.04, *p* = 0.005). All data above indicate that B-cell-related humoral immunity was enhanced in NB patients.

### γδ TFH cells was positively correlated with serum total IgG and plasma cells in NB patients

γδ TFH cells secrete IL-4 and IL-10, both of which could regulate B-cell proliferation, differentiation, and class switching [13, 14]. We further analyzed the relationship between γδ TFH cells and B cells in NB patients.

**Fig. 5:**
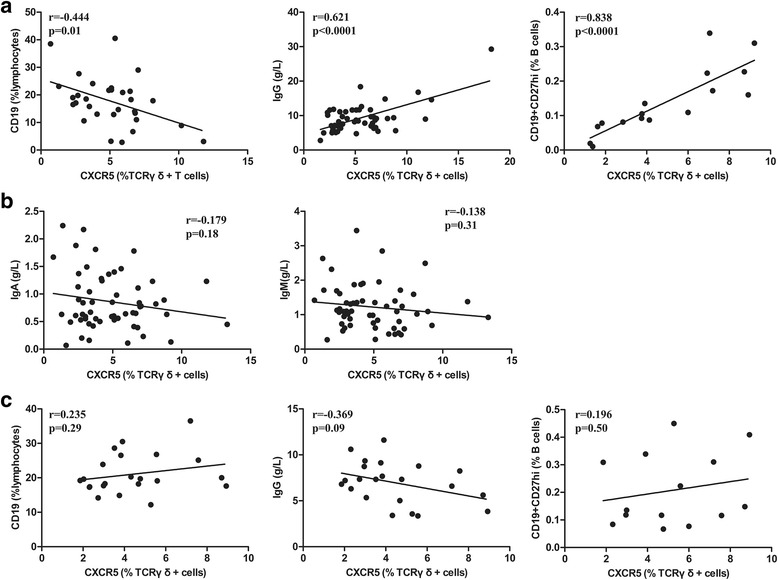
The frequency of γδ TFH cells is positively correlated to the serum total IgG level and CD19^+^CD27^hi^ plasma cells in NB patients. **a** Correlation of CXCR5^+^ γδT cells with CD19^+^ B cells, serum total IgG level or CD19^+^CD27^hi^ plasma cells in NB patients. Each dot represents one individual. **b** Correlation of CXCR5^+^ γδT cells with CD19^+^ B cells, serum total IgG level or CD19^+^CD27^hi^ plasma cells in healthy control. Each dot represents one individual. **c** Correlation of CXCR5^+^ γδT cells with serum total IgA level or serum total IgM level in NB patients. Each dot represents one individual

In peripheral blood of NB patients, CXCR5^+^ γδT cells were found negatively correlated with CD19^+^ B cells (*r* = -0.444, *p* = 0.01, Fig. 5a). Next, we investigated the correlation between the percentage of CXCR5^+^ γδT cells and serum total IgG, IgA or IgM levels in NB patients. As shown in Fig. 5a, there was a positive correlation between the frequency of CXCR5^+^ γδT cells and serum total IgG level (*r* = 0.621, *p* < 0.0001, Fig. 5a), but no correlation was found with serum total IgA or IgM level (IgA, *r* = -0.179, *p* = 0.18; IgM, *r* = -0.138, *p* = 0.31, Fig. 5b). Moreover, CXCR5^+^ γδT cells were found positively correlated with CD19^+^CD27^hi^ plasma cells (*r* = 0.838, *p* < 0.0001, Fig. 5a). However, no correlation was found between CXCR5^+^ γδT cells and CD19^+^ B cells, serum total IgG level or CD19^+^CD27^hi^ plasma cells in healthy control (CD19^+^ B cells, *r* = 0.235, *p* = 0.29; IgG, *r* = -0.369, *p* = 0.09; plasma cells, r = 0.196, *p* = 0.50, Fig. 5c). Together, these data suggest that these γδ TFH cells are associated with antibody-mediated immune responses of the NB patients.

## Discussion

It is clear that γδ TFH cells is responsible, at least in part, for support of B cell functions [15–17]. Comparatively, little is known about their role in antibody-mediated immune responses in NB patients. In the present study, we observed a significant expanded γδ TFH cells in patients diagnosed with NB compared with healthy controls. γδ TFH cells secreted IL-4 and IL-10 was increased in NB patients. Moreover, we also observed an increase in serum total IgG level and enhanced plasma cells in NB individuals. Furthermore, we demonstrated that γδ TFH cells were associated with the serum total IgG level and CD19^+^CD27^hi^ plasma cells in NB patients.

Previous studies have shown that in vitro differentiated TFH-like γδT cells have a predominant central memory and distinctively express CD40L, ICOS and CXCR5 [16, 18]. Nadia et al. demonstrated that most of circulating CXCR5^+^ γδT cells have a central memory phenotype with down-regulation of the activation markers (CD25, HLA-DR) and costimulatory molecules (CD40L, ICOS) in healthy volunteers. We showed that most γδ TFH cells have a naive phenotype in NB patients. Furthermore, most γδ TFH cells express both activation (CD25, CD69 and HLA-DR) and costimulatory (CD40L) molecules, but do not express ICOS in NB patients. Our results might indicate a specific phenotype of γδ TFH cells in peripheral blood of tumor patients.

Recent investigations suggest that production of great amounts of cytokines from γδT cells may influence B cell responses in humoral immunity [17, 19]. In contrast to CD4 αβ TFH cells, γδ TFH cells do not produce IL-21, but secrete IL-4 and IL-10 upon Ag stimulation in vitro [7, 16]. Studies in mouse model demonstrated that γδT cells affect IL-4 production and B-cell activation [15]. Gascan H et al. demonstrated that in vitro activated γδ TFH cells induce B cell activation and Ig isotype switching in the presence of IL-4 [20]. In our study, we found that γδ TFH cells in NB patients secreted higher level of IL-4 and IL-10 compared with that in healthy controls. This may explain why serum total IgG level was significantly increased in NB patients in the context of expanded plasma cells. Whether IL-4 and IL-10 could directly influence antibody-mediated immune responses was not addressed in our current issue and still need further investigation.

Plasma cells, the sole producers of immunoglobulins, are critical for an effective humoral immunity [12, 21]. Terminally differentiated plasma cells express relatively fewer common pan-B cell markers, such as CD19 and CD20. This may explain why the frequency of CD19^+^CD27^hi^ plasma cells were increased in NB individuals while the frequency of CD3^−^CD19^+^ B cells decreased in NB patients.

## Conclusion

Our present study demonstrated that γδ TFH cells are associated with antibody-mediated immune responses in NB patients. Our findings highlight the role of γδ TFH cells-mediated immune responses in NB patients and might provide a potential therapeutic target for the treatment of NB patients.

## Abbreviations

TFH cells: Follicular helper T cells
NKT: Natural killer T cells
NB: Neuroblastoma
IL-2: Interleukin 2
IL-4: Interleukin 4
IL-10: Interleukin 10
IFNγ: Interferon gamma
